# From balance to imbalance: disruption of plasma glutathione concentration in micropapillary thyroid carcinoma

**DOI:** 10.1186/s13044-024-00204-9

**Published:** 2024-07-01

**Authors:** Fatemeh Eskandari, Mehdi Hedayati, S. Mohammad Tavangar, Farnaz Rezaei, Afsaneh Khodagholipour, S. Adeleh Razavi

**Affiliations:** 1 Department of Stem Cell and Regenerative Medicine, National Institute of Genetic Engineering and Biotechnology, Tehran, Iran; 2 Cellular and Molecular Endocrine Research Center, Research Institute for Endocrine Sciences, Shahid Beheshti University of Medical Sciences, No 23, Shahid Arabi St. Yemen St. Velenjak, PO Box: 1985717413, Tehran, Iran; 3 Department of Pathology, Shariati Hospital, School of Medicine, Tehran University of Medical Sciences, Tehran, Iran; 4 Chronic Diseases Research Center, Endocrinology and Metabolism Population Sciences Institute, Tehran University of Medical Sciences, Tehran, Iran; 5 Department of Cellular and Molecular Biology, Faculty of Advanced Sciences and Technology, Tehran Medical Sciences Branch, Islamic Azad University, Tehran, Iran; 6 Department of Anesthesia, Faculty of Paramedical, Qom University of Medical Sciences, Qom, Iran

**Keywords:** Papillary thyroid cancer, Oxidative stress, Antioxidant system, Glutathione, Reduced/oxidized glutathione ratio

## Abstract

**Background:**

Despite the presence of evidence that establishes a strong correlation between oxidative stress and thyroid cancer, there exists a scarcity of research that investigates the specific role of glutathione as an important antioxidant in this particular context. The objective of this study was to assess the altered balance of oxidative stress in cases of thyroid cancer, which includes both papillary thyroid carcinoma (PTC) and micro PTC (mPTC), by examining and comparing the total antioxidant capacity (TAC), total oxidant status (TOS), oxidative stress index (OSI), reduced glutathione (GSH), oxidized glutathione (GSSG), and GSSG/GSH ratio with those of individuals diagnosed with multinodular goiter (MNG) as well as Healthy subjects.

**Materials and methods:**

Plasma samples were collected from 92 patients (23 mPTC, 23 PTC, 23 MNG, 23 Healthy). The levels of TAC, TOS, GSH, and GSSG were measured using a commercial assay kits, and the OSI and GSSG/GSH ratio were calculated for each sample. Statistical analyses were performed to compare the oxidative stress between the groups.

**Results:**

The plasma levels of TOS were significantly higher in the mPTC, PTC, and MNG groups compared to the Healthy individuals (*p* < 0.05). The OSI in the mPTC and PTC groups showed a significant increase compared to the Healthy group (*p* < 0.05). The levels of GSH in mPTC and PTC were markedly lower compared to the Healthy subjects (*p* < 0.01). Interestingly, the concentration of GSH in mPTC was found to be considerably lower than in PTC and MNG patients (*p* < 0.01).

**Conclusion:**

These findings indicate that GSH may be a useful biomarker for evaluating oxidative stress and antioxidant system status in patients with PTC, especially mPTC. Low levels of GSH may indicate increased levels of oxidative stress, which may contribute to the development and progression of mPTC to PTC.

## Introduction

The occurrence of malignancy in the endocrine tissue of the human body is rare. Nevertheless, the thyroid gland exhibits the highest prevalence of malignancy and is responsible for approximately 2.9% of the overall cancers in the human population [1]. Thyroid cancer encompasses several subtypes, with papillary thyroid carcinoma (PTC) being the most frequently observed [2]. PTC includes a subset known as micro PTC (mPTC), which has indicated a rise in incidence as a result of the progress made in diagnostic techniques centered around ultrasound. The term mPTC was initially introduced by the World Health Organization (WHO) in 1989 [3] and comprises of PTC tumors that measure less than or equal to 1 cm (10 mm) in size [4–6]. In the 8th edition of the TNM classification, mPTC is classified as pT1a [7, 8]. The 2022 WHO classification of thyroid tumors advised against categorizing these PTCs solely as “mPTC” without any supplementary subtype information [9, 10].

Although the prognosis for mPTC and PTC is typically promising when identified in its early stages, there exist certain circumstances in which the disease might progress and present difficulties in relation to medical intervention [11]. Antioxidants are significant molecules that assume a pivotal function in protecting the cells against oxidative stress. Although they are not commonly employed as direct cancer biomarkers, alterations in the levels of antioxidants can indirectly manifest the presence, development or progression of cancer [12]. Measuring the total antioxidant capacity (TAC) and total oxidant status (TOS) of individuals diagnosed with mPTC or PTC provides insights into the overall oxidative stress levels and aids in understanding its role in the pathogenesis and therapeutic strategies of this medical condition.

Total antioxidant defense system consists of a variety of molecules that work harmoniously together to counteract the harmful effects of reactive oxygen species (ROS) to maintain cellular oxidative balance. These molecules participate in the process of neutralizing ROS, and repairing oxidative damage. The antioxidant defense system is composed of many different components, including enzymatic antioxidants, non-enzymatic antioxidants, metal-binding proteins, and other small molecules with antioxidant activity. Enzymatic antioxidants include superoxide dismutase, catalase and glutathione peroxidase, and non-enzymatic antioxidants include vitamin C, vitamin E, melatonin, and glutathione [13, 14].

Glutathione is an endogenous compound plays a pivotal role as a crucial scavenger of ROS and free radicals, thereby exerting a significant impact on the maintenance of cellular health. This tripeptide is formed by three amino acids, namely cysteine, glycine, and glutamate. Glutathione exists in two distinct form of reduced glutathione (GSH) and oxidized glutathione (GSSG) [15]. The latter form, GSSG, represents a disulfide configuration of glutathione, and is thereby employed as a diagnostic indicator for oxidative stress. GSH has the capability to donate an electron to lower the reactivity of other molecules, thereby transforming them into forms that are less reactive or non-toxic [16]. Optimal cellular function is strongly dependent on the balance between oxidation and reduction reactions. The ratio of GSSG to GSH functions as an indicator illustrating the redox state within the cellular environment and is importance in ensuring the maintenance of optimal cellular functionality [17, 18].

Despite the existence of substantial evidence establishing a connection between oxidative stress and thyroid cancer, a deficiency of investigations exists that explore the specific role of glutathione in this specific context. Attaining a thorough understanding of the imbalance between oxidants and the ability of cells to scavenge these harmful molecules has the potential to enhance comprehension of possible mechanisms and introduce prospective targets for the diagnosis or treatment of thyroid cancer.

The main goal of this study was to assess the altered oxidative balance in cases of thyroid cancer through the examination of TAC, TOS, oxidative stress index (OSI), GSH, GSSG, and GSSG/GSH ratio. We conducted the analysis on a group of individuals afflicted with mPTC or PTC, and compared their overall (TAC, TOS, OSI) and particular (GSH, GSSG, GSSG/GSH) oxidative profiles with those of multinodular goiter (MNG) and healthy individuals. An enhanced comprehension of the molecular mechanisms responsible for the development and progression of mPTC and PTC could yield novel approaches for the prevention, early identification, and management of this malignancy.

## Materials and methods

### Study population

All patients referred to Laleh Hospital in Tehran, Iran, for total thyroidectomy between January 2023 and October 2023 were initially enrolled in the research study. Plasma specimens were collected from the subjects before their surgical interventions. Following the surgeries, patients were categorized according to their histopathological findings. The primary criterion for inclusion in the research cohorts was the confirmation of mPTC, PTC, or MNG through postoperative histopathological assessments conducted by experienced pathologists. The presence of malignancy of any form (including thyroid malignancies other than PTC) or chronic illnesses in the subjects constituted the exclusion criteria. Another additional criterion for exclusion from the research was the administration of medications, radiation therapy, or chemotherapy.

The Healthy group comprised individuals devoid of antecedent chronic or non-chronic ailments, specifically thyroid-related disorders, who self-assessed their health status as optimal based on health questionnaires. This cohort exhibited normal thyroid stimulating hormone (TSH) levels and, as confirmed by sonographic assessment, lacked any discernible thyroid nodules.

Ultimately, this case-control study consisted of 92 participants divided into four groups: 23 individuals with mPTC, 23 individuals with PTC, 23 individuals with MNG, and 23 Healthy subjects.

The study was approved by the Institutional Review Board and Ethics Committee of the Research Institute for Endocrine Sciences. All participants provided written informed consent prior to the blood collection.

### Sample collection

Before the surgery, the plasma was acquired from the peripheral vein of all the individuals following an overnight fasting period, and it was collected utilizing lithium heparin plasma tube, which was subsequently subjected to centrifugation at 4000 rpm for a duration of 15 min at a temperature of 4 °C. Subsequently, the sample was divided into Eppendorf microtubes and stored at a temperature of − 80 °C until the time of analysis.

### Measurement of TAC and TOS

Plasma levels of TAC and TOS were measured using commercial assay kits (ZellBio GmbH, Germany) according to the manufacturer’s instructions. The assays utilized a colorimetric method to quantify the TAC or TOS levels based on the intensity of the generated signal which was read spectrophotometrically at 460–490 nm or 560 nm respectively.

The TAC kit had a measurement range of 0.125-2 mM, with a sensitivity of 0.1 mM. The intra-assay precision was reported as a coefficient of variation (CV) < 3.4%, while the inter-assay precision was reported as CV < 4.2%. The TOS kit exhibited a detection range of 1.25-20 µM, with a sensitivity of 0.5 µM. In terms of precision, the intra-assay variation was reported as CV < 4.2%, while the inter-assay variation was reported as CV < 6.9%.

### Measurement of OSI

The OSI value was determined by employing the subsequent formula: OSI (expressed in percentage unit) = [(TOS, measured in µM)/(TAC, measured in µM)×100] [19].

### Measurement of GSH and GSSG

Plasma levels of GSH and GSSG were measured using commercial assay kits (ZellBio GmbH, Germany) according to the manufacturer’s instructions. The assays employed a colorimetric method to measure the GSH and GSSG levels by assessing the intensity of the signal produced, which was subsequently analyzed spectrophotometrically at 412 nm.

The GSH assay kit offered a measurement range of 0.03-1 mM, with a sensitivity of 0.01 mM. It demonstrated intra-assay precision, with a CV about 3.1%. Similarly, the inter-assay precision was also reported as CV ~ 4.7%. On the other hand, the GSSG assay kit had a detection range of 0.03 to 1 mM, with a sensitivity of 0.01 mM. The intra- and inter assay variation was reported as CV ~ 4.2% and CV ~ 4.9% respectively.

The GSSG/GSH ratio was calculated for each sample as an indicator of the redox balance within a biological system.

### Statistical analysis

Firstly, an assessment was made of the normal distribution of the data related to the examined groups. Parametric tests (unpaired t-test, One-Way ANOVA and the Tukey post hoc test) were employed in order to determine any significantly differences between the normally distributed data, while non-parametric tests (Mann-Whitney test) were utilized for data that did not conform to the normal distribution. Receiver operating characteristic (ROC) curve analysis was employed for the variables that exhibited notable distinctions amongst the investigated groups under investigation. The potency of the significant variables as risk factors was assessed through the utilization of the binary logistic regression model. The statistically significance level for all tests was set at 0.05. GraphPad Prism 8.0.1 (GraphPad Software, San Diego, CA, USA) and MedCalc 19.2 (MedCalc Software Ltd., Ostend, Belgium) were utilized in order to perform the statistical analyses.

## Results

### Demographic and pathological characteristics

Demographic and pathological characteristics of the study participants are shown in Table 1. There were no significant disparities in the age of the subjects when comparing mPTC to PTC, mPTC to MNG, and PTC to MNG cohorts. However, a significant age discrepancy was observed in the mPTC vs. Healthy (*P* = 0.0208), PTC vs. Healthy (*P* = 0.0049), and MNG vs. Healthy (*P* = 0.0009) groups. No significant variations in gender were found within any of the cohorts.

**Table 1:** Demographic and pathological characteristics of the study participants

Parameter	mPTC (*n* = 23)	PTC (*n* = 23)	MNG (*n* = 23)	Healthy (*n* = 23)
**Age (years)** Mean ± SD	43.30 ± 12.52	45.65 ± 11.44	47.00 ± 10.86	34.61 ± 13.07
**Gender**				
Female	19	18	19	19
Male	4	5	4	4
**Histology Type**				
Classical Variant	21	19		
Follicular Variant	2	4		
**Tumor size (cm)**				
Mean ± SD	0.61 ± 0.22	1.90 ± 1.18		
**Tumor site**				
Right lobe	12	16		
Left lobe	8	5		
Isthmus	3	2		
Tumor focality*				
Unifocal	14	11		
Bifocal	8	5		
Multifocal	1	7		
**Encapsulated tumor**				
Absent	22	11		
Present	1	12		
**Extrathyroidal extension**				
Absent	23	21		
Present	0	2		
**Lymphovascular invasion**				
Absent	14	7		
Present	9	16		
**Lymph node metastasis**				
Absent	18	9		
Present	5	14		
**TNM stage**				
**I/II**	23	16		
**III/IV**	0	7		

* The information provided is for the most prominent tumor

MNG, multinodular goiter; mPTC, micro papillary thyroid carcinoma; PTC, papillary thyroid carcinoma; TNM, Tumor-Node-Metastasis staging system

### Plasma levels of TAC, TOS, and OSI in studied groups

**Fig. 1:**
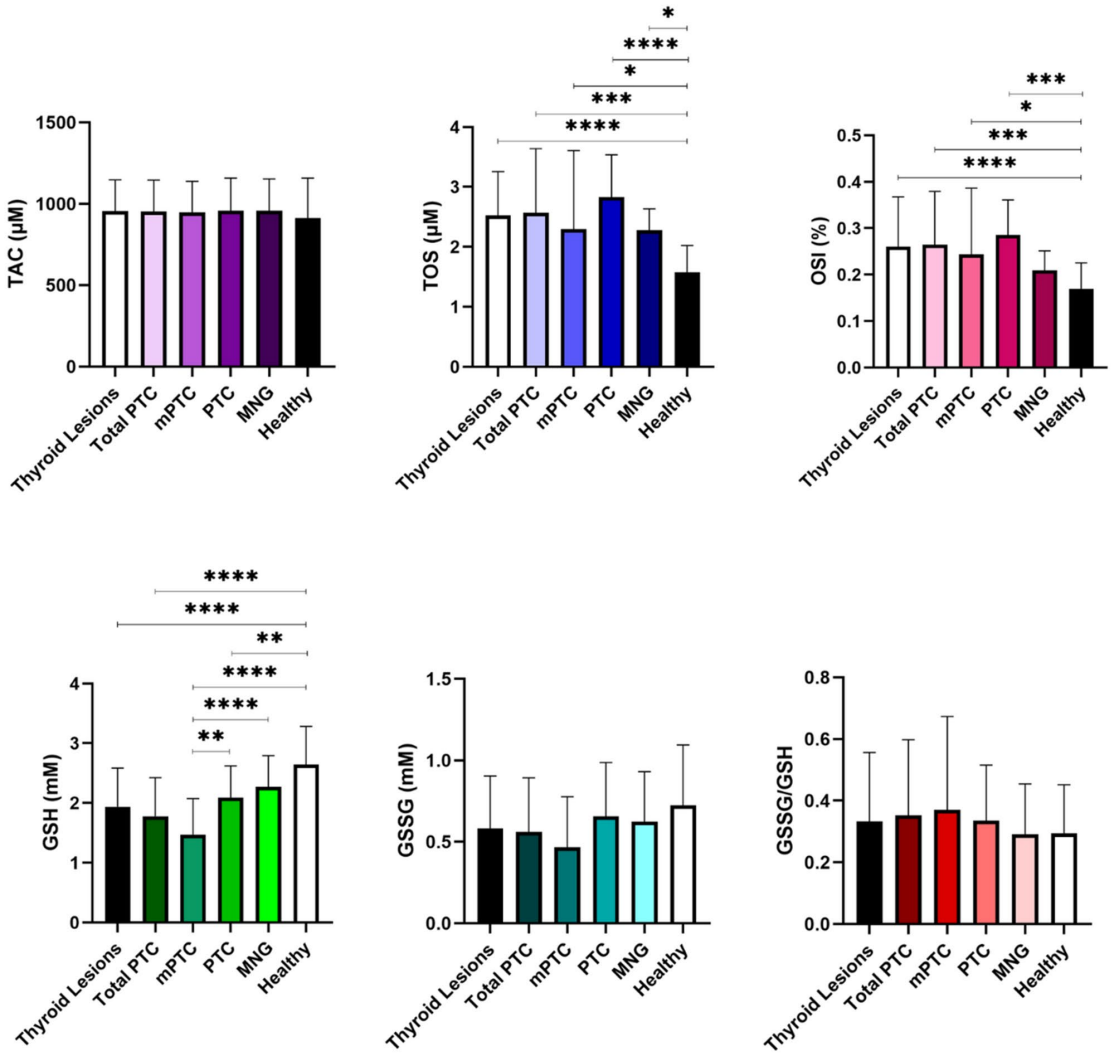
Plasma levels of TAC, TOS, OSI, GSH, GSSG, and GSSG/GSH ratio in patients and Healthy subjects. The p-value < 0.05 is flagged with one star (*), the p-value < 0.01 is flagged with 2 stars (**), the p-value < 0.001 is flagged with three stars (***), and the p-value < 0.0001 is flagged with 4 stars (****)

The concentration of TAC in the patients group was as follows: (i) Thyroid Lesions (mPTC + PTC + MNG) = 954.37 ± 192.74 µM, (ii) Total PTC (mPTC + PTC) = 952.84 ± 193.29 µM, (iii) mPTC = 948.22 ± 190.11 µM, (iv) PTC = 957.67 ± 200.91 µM, (v) MNG = 957.35 ± 195.97 µM, and (vi) Healthy = 912.89 ± 245.01 µM. TAC did not show any significant variation among the studied groups (Fig. 1; Table 2).

**Table 2:** Comparison of plasma levels of TAC, TOS, and OSI in the study population

Groups	TAC		TOS		OSI	
	Significantly different	*P*-Value	Significantly different	*P*-Value	Significantly different	*P*-Value
Thyroid Lesions vs. Healthy	No	0.4082^a^	Yes	< 0.0001^a^	Yes	< 0.0001^c^
Total PTC vs. Healthy	No	0.4647^a^	Yes	0.0001^a^	Yes	0.0008^a^
mPTC vs. PTC	No	0.9988^b^	No	0.1329^b^	No	0.4454^b^
mPTC vs. MNG	No	0.9988^b^	No	0.9998^b^	No	0.6714^b^
mPTC vs. Healthy	No	0.9400^b^	Yes	0.0245^b^	Yes	0.0495^b^
PTC vs. MNG	No	> 0.9999^b^	No	0.1425^b^	No	0.0717^b^
PTC vs. Healthy	No	0.8898^b^	Yes	< 0.0001^b^	Yes	0.0006^b^
MNG vs. Healthy	No	0.8886^b^	Yes	0.0443^b^	No	0.5840^b^

^a^ P-values are from the unpaired t-test. A P-value of < 0.05 was considered statistically significant

^b^ P-values are from the One-Way ANOVA and the Tukey post hoc test. A P-value of < 0.05 was considered statistically significant

^c^ P-value is from the Mann-Whitney test. A P-value of < 0.05 was considered statistically significant

mPTC, micro papillary thyroid carcinoma; PTC, papillary thyroid carcinoma; MNG, multinodular goiter; TAC, total antioxidant capacity; TOS, total oxidant status; OSI, oxidative stress index

The plasma concentration of TOS was: (i) Thyroid Lesions (mPTC + PTC + MNG) = 2.48 ± 0.93 µM, (ii) Total PTC (mPTC + PTC) = 2.57 ± 1.07 µM, (iii) mPTC = 2.30 ± 1.31 µM, (iv) PTC = 2.83 ± 0.70 µM, (v) MNG = 2.28 ± 0.36 µM, and (vi) Healthy = 1.58 ± 0.45 µM. The levels of TOS in all three thyroid lesions (mPTC, PTC, MNG) exhibited significant differences when compared to the Healthy group (Fig. 1; Table 2).

The index of OSI (%) in the plasma was: (i) Thyroid Lesions (mPTC + PTC + MNG) = 0.25 ± 0.10, (ii) Total PTC (mPTC + PTC) = 0.26 ± 0.11, (iii) mPTC = 0.24 ± 0.14, (iv) PTC = 0.29 ± 0.08, (v) MNG = 0.21 ± 0.04, and (vi) Healthy = 0.17 ± 0.06. The OSI in mPTC and PTC patients demonstrated significant disparities in comparison to the Healthy group (Fig. 1; Table 2).

### Plasma levels of GSH, GSSG, and GSSG/GSH ratio in studied groups

The patients group showcased distinct concentrations of GSH as follows: (i) Thyroid Lesions (mPTC + PTC + MNG) = 1.94 ± 0.65 mM, (ii) Total PTC (mPTC + PTC) = 1.78 ± 0.64 mM, (iii) mPTC = 1.47 ± 0.61 mM, (iv) PTC = 2.09 ± 0.53 mM, (v) MNG = 2.27 ± 0.52 mM, and (vi) Healthy = 2.64 ± 0.64 mM. The studied groups, which encompassed Thyroid Lesions vs. Healthy, Total PTC vs. Healthy, mPTC vs. Healthy, and PTC vs. Healthy, exhibited significant disparities in the levels of GSH. Furthermore, the differing levels of GSH were observed not only between mPTC vs. PTC but also between mPTC vs. MNG, indicating their statistical significance (Fig. 1; Table 3).

**Table 3:** Comparison of plasma levels of GSH, GSSG, and GSSG/GSH ratio in the study population

Groups	GSH		GSSG		GSSG/GSH	
	Significantly different	*P*-Value	Significantly different	*P*-Value	Significantly different	*P*-Value
Thyroid Lesions vs. Healthy	Yes	< 0.0001^a^	No	0.1014^a^	No	0.4724^a^
Total PTC vs. Healthy	Yes	< 0.0001^a^	No	0.0864^a^	No	0.3354^a^
mPTC vs. PTC	Yes	0.0026^b^	No	0.2243^b^	No	0.9456^b^
mPTC vs. MNG	Yes	< 0.0001^b^	No	0.4092^b^	No	0.6231^b^
mPTC vs. Healthy	Yes	< 0.0001^b^	No	0.0635^b^	No	0.6516^b^
PTC vs. MNG	No	0.7208^b^	No	0.9880^b^	No	0.9056^b^
PTC vs. Healthy	Yes	0.0093^b^	No	0.9175^b^	No	0.9219^b^
MNG vs. Healthy	No	0.1474^b^	No	0.7796^b^	No	> 0.9999^b^

^a^ P-values are from the unpaired t-test. A P-value of < 0.05 was considered statistically significant

^b^ P-values are from the One-Way ANOVA and the Tukey post hoc test. A P-value of < 0.05 was considered statistically significant

mPTC, micro papillary thyroid carcinoma; PTC, papillary thyroid carcinoma; MNG, multinodular goiter; GSH, reduced glutathione; GSSG, oxidized glutathione

The plasma concentration of GSSG quantified the following values: (i) Thyroid Lesions (mPTC + PTC + MNG) = 0.58 ± 0.32 mM, (ii) Total PTC (mPTC + PTC) = 0.56 ± 0.33 mM, (iii) mPTC = 0.47 ± 0.31 mM, (iv) PTC = 0.66 ± 0.33 mM, (v) MNG = 0.62 ± 0.31 mM, and (vi) Healthy = 0.72 ± 0.37 mM. The levels of GSSG were not significantly different in any of the investigated groups (Fig. 1; Table 3).

The plasma exhibited a GSSG/GSH ratio of: (i) Thyroid Lesions (mPTC + PTC + MNG) = 0.33 ± 0.22, (ii) Total PTC (mPTC + PTC) = 0.38 ± 0.32, (iii) mPTC = 0.37 ± 0.30, (iv) PTC = 0.34 ± 0.18, iv) MNG = 0.29 ± 0.16, and vi) Healthy = 0.29 ± 0.16. The GSSG/GSH values were not significantly different among the studied groups (Fig. 1; Table 3).

### Oxidative stress parameters in PTC subgroups

Oxidative stress parameters (TAC, TOS, OSI, GSH, GSSG, GSSG/GSH) in the Total PTC group were evaluated with respect to demographic and pathological features with adequate sample sizes. The findings from this analysis indicated the absence of any statistically significant differences among the demographic and pathological subgroups. A comprehensive account of this analysis can be found in Table 4.

**Table 4:** Comparison of plasma levels of oxidative stress parameters in the Total PTC subgroups

Groups	TAC		TOS		OSI	
	Significantly different	*P*-Value^a^	Significantly different	*P*-Value^a^	Significantly different	*P*-Value^a^
< 45 vs. ≥ 45 years	No	0.5288	No	0.3365	No	0.9495
Females vs. males	No	0.3551	No	0.4871	No	0.4159
Absence of lymphovascular invasion vs. presence	No	0.8325	No	0.3960	No	0.3689
Absence of lymph node metastasis vs. presence	No	0.5644	No	0.5406	No	0.7442
**Groups**	**GSH**		**GSSG**		**GSSG/GSH**	
	Significantly different	*P*-Value^a^	Significantly different	*P*-Value^a^	Significantly different	*P*-Value^a^
< 45 vs. ≥ 45 years	No	0.7546	No	0.8158	No	0.4329
Females vs. males	No	0.8507	No	0.0571	No	0.1082
Absence of lymphovascular invasion vs. presence	No	0.5072	No	0.4412	No	0.6722
Absence of lymph node metastasis vs. presence	No	0.3225	No	0.4070	No	0.7087

^a^ P-values are from the unpaired t-test. A P-value of < 0.05 was considered statistically significant

### Diagnostic value of GSH

**Fig. 2:**
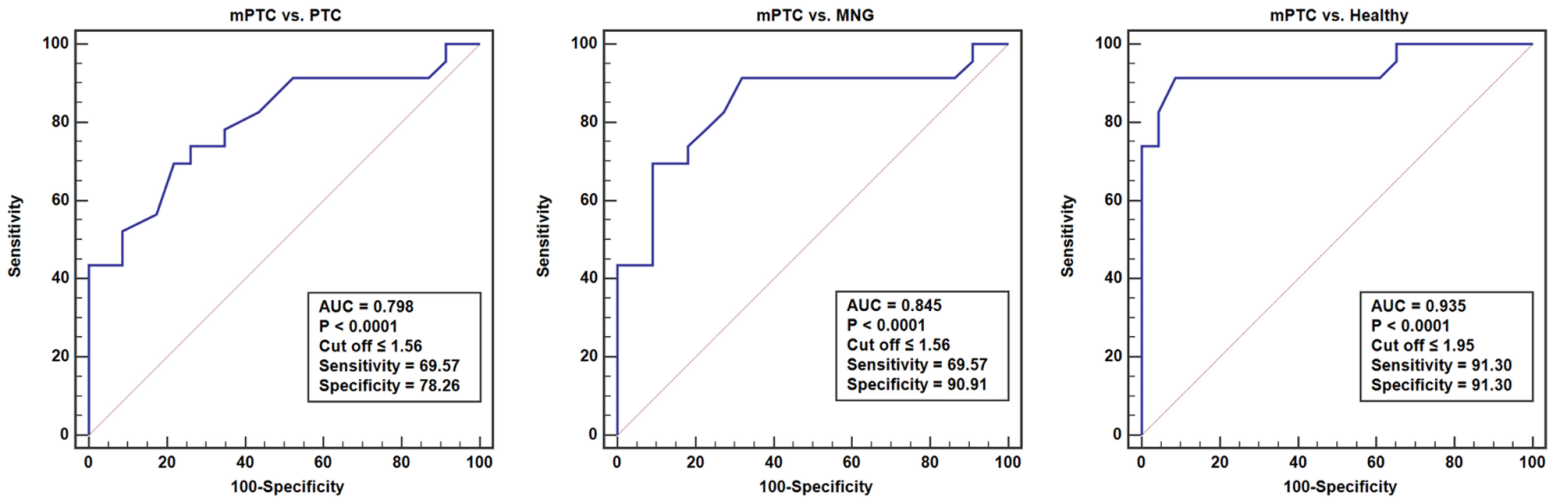
The ROC curve analyses of GSH marker for distinguishing mPTC from PTC, MNG, and Healthy subjects

**Fig. 3:**
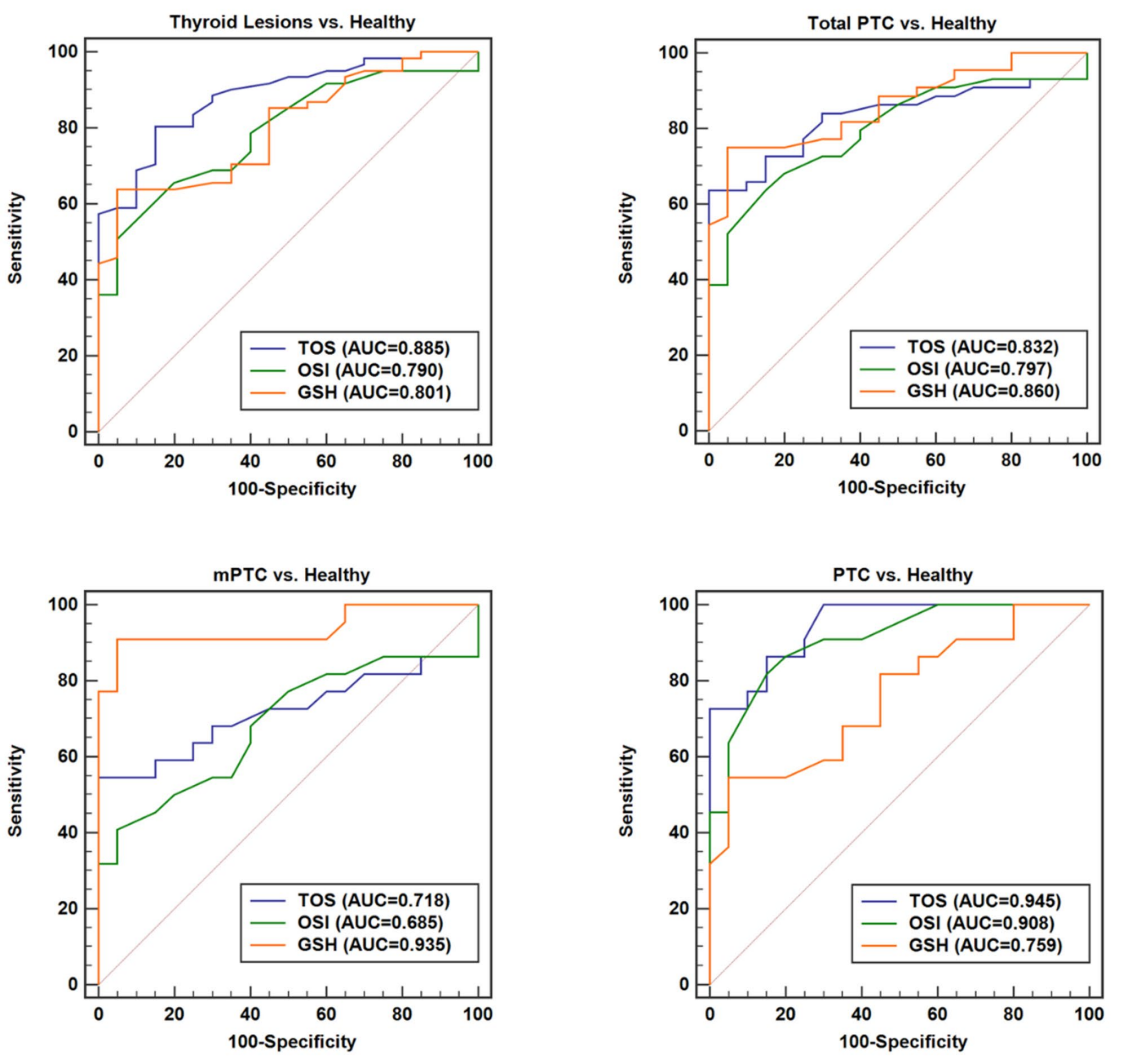
The ROC curve analyses of TOS, OSI, and GSH markers for distinguishing thyroid patients groups from Healthy subjects

The diagnostic value of GSH was determined using the ROC curve analysis. GSH exhibited the capability to diagnose Thyroid Lesions, Total PTC, PTC, and mPTC in comparison to Healthy samples. However, the best results were obtained for the diagnosis of mPTC from Healthy subjects. On the other hand, this marker had the ability to distinguish mPTC from PTC as well as mPTC from MNG (Fig. 2). The ROC curve was also utilized to evaluate the diagnostic significance of TOS and OSI markers (Fig. 3). TOS and OSI showed the ability to distinguish Thyroid Lesions, Total PTC, PTC, and mPTC from Healthy individuals; however, the best result was obtained for distinguishing PTC from Healthy subjects (TOS: AUC = 0.94, *P* < 0.0001, Cut off > 2.36, Sensitivity = 73.91, Specificity = 100.00 and OSI: AUC = 0.90, *P* < 0.0001, Cut off > 0.22, Sensitivity = 81.82, Specificity = 85.00).

### Risk factor value of GSH

Logistic regression analysis employed as a statistical method to explore the potential relationship between reduced levels of GSH and the occurrence of mPTC or PTC. The findings revealed that a decrease in the concentration of GSH is linked to an elevated likelihood of developing mPTC and PTC in comparison to Healthy individuals. The logistic regression outputs are illustrated in Table 5.

**Table 5:** Logistic regression analysis of GSH in mPTC and PTC groups compared to the Healthy controls

Groups	B	S.E.	*P*-value	Odds Ratio (OR)	95% CI for OR
**mPTC vs. Healthy**	-3.214	0.971	**0.001**	**0.040**	0.006–0.270
**mPTC vs. Healthy** (adjusted for age and gender)	-3.237	0.990	**0.001**	**0.039**	0.006–0.273
**PTC vs. Healthy**	-1.638	0.611	**0.007**	**0.194**	0.059–0.643
**PTC vs. Healthy** (adjusted for age and gender)	-1.750	0.676	**0.010**	**0.174**	0.046–0.654

mPTC, micro papillary thyroid carcinoma; PTC, papillary thyroid carcinoma

## Discussion

The mechanisms by which oxidative stress may contribute to the development of thyroid cancer have not yet been fully elucidated. However, it appears that the disruption of intracellular redox systems can potentially lead to an overabundance of oxidants, which in turn may play a role in the molecular pathogenesis of thyroid cancer. ROS have the capacity to inflict damage to DNA and induce genetic alterations. Genetic alterations, such as *BRAF*, *RAS*, *PIK3CA*, and *PTEN* mutations, serve as the driving force behind the activation of the MAPK and PI3K/Akt pathways, which represent the fundamental mechanism in the development of thyroid cancer. Chemicals such as bisphenol AF and diethylhexylphthalate, significantly associated with thyroid malignancies, may also be associated with oxidative stress [28, 29]. In this particular investigation, we assessed the overall (TAC, TOS, OSI) and particular (GSH, GSSG, GSSG/GSH) oxidative status of patients diagnosed with PTC. The findings of this study revealed that the TOS status and OSI value are significantly different between studied groups. OSI may be a valuable index to serve as a biomarker for evaluating the overall oxidative stress in individuals afflicted with PTC. Our discoveries revealed that the plasma levels of GSH were notably lower among the mPTC, and PTC cohorts in comparison to the Healthy group, suggesting a diminished capacity for antioxidation in these patients. 

The investigation of the condition of oxidative stress in thyroid cancer has been addressed in an influential study conducted by Wang et al. [20]. Within this research, TAC, TOS, and OSI were examined in a cohort of 82 individuals with thyroid cancer, 56 patients with benign conditions, and 50 healthy subjects. The findings from the study revealed that the levels of serum TAC were notably lower in patients with thyroid cancer compared to the controls. Conversely, the levels of serum TOS and OSI values were significantly higher in the cancer patients [20]. A separate study was conducted to compare the serum levels of oxidative stress in PTC patients diagnosed with angioinvasion and metastasis with those without angioinvasion and metastasis. This investigation revealed a significant reduction in TAC, while no change was observed in TOS [21]. A recent investigation examined the oxidative homeostasis in patients diagnosed with PTC who were assigned to receive radioiodine (RAI) treatment. The study compared the values of TAC and TOS in these patients with those of very low-risk PTC patients who were not assigned for RAI treatment. This study disclosed that among the examined cohort, the serum TOS concentration showed a significantly elevated level, while the TAC concentration demonstrated a markedly reduced level compared to the control group [22].

Two studies have been undertaken to explore the condition of oxidative stress in tissues impacted by thyroid cancer. One of them demonstrated that while TOS did not exhibit a significant difference between PTC tissues and normal/goiter tissues, the TAC values in PTC were significantly higher compared to the values in goiter and normal tissues [23]. The other one showed that the level of TAC was noticeably decreased in medullary thyroid carcinoma (MTC) and follicular thyroid carcinoma (FTC) subtypes compared to the control group. Conversely, the level of TOS was significantly increased in the MTC group when compared to both the control group and the group with benign nodules. Furthermore, the OSI exhibited a considerable elevation in both MTC and FTC subtypes [24].

In our study, no significant difference in TAC was observed between different groups. However, the levels of TOS were notably elevated in all groups when compared to the Healthy subjects. Similarly, OSI exhibited higher values in most groups in comparison to the Healthy individuals. Nevertheless, TOS proved to be a more effective parameter for distinguishing between mPTC, PTC, and MNG. It is important to note that the conflicting findings exist in different studies may be due to the variability in factors such as age, gender, lifestyle, and comorbidities among the patient cohorts. On the other hand, the heterogeneous nature of PTC characterized by divers genetic alterations may contribute to differing results.

A valuable investigation on colorectal carcinoma (CRC) has been carried out with regard to the analysis of the serum levels of GSH, GSSG, and their ratio in 80 individuals diagnosed with CRC and 60 healthy individuals. The findings demonstrated a significant reduction in GSH levels and a significant increase in GSSG levels as well as the GSSG/GSH ratio among patients diagnosed with CRC. This research has introduced the GSSG/GSH ratio as an innovative tumor marker for the identification and monitoring of CRC [25]. In the year 2020, an investigation was carried out on the antioxidant defense condition in individuals diagnosed with Hashimoto’s thyroiditis. The findings of this examination revealed a significantly reduction in the serum GSH level when compared to the control cohort [26]. In our recent investigation, utilizing the metabolomics methodology, it was observed that GSH is one of the metabolites manifests a decreased plasma level in PTC compared to that of healthy individuals [27].

In this particular study, we have made observations that indicated a significantly decline in the GSH level amongst individuals diagnosed with PTC and mPTC as compared to Healthy individuals. Conversely, the decrease in GSH level in mPTC patients was also found to be statistically significant when compared to those of PTC and MNG. Given that the concentration of GSH in mPTC differed from that in all three categories of PTC, MNG, and Healthy individuals, this particular metabolite can be introduced as a diagnostic biomarker for mPTC, in conjunction with sonographic techniques. However, the clinical role of GSH as a diagnostic tumor marker in mPTC and PTC should be studied more extensively with a large sample size. Such studies exploring the relationship between oxidative stress parameters and thyroid cancer can provide valuable insights into the potential involvement of oxidative stress in the pathogenesis of this disease, however, it is important to note that the nature of this association remains correlative and does not imply a causal relationship. Further research is needed to elucidate the mechanistic underpinnings of these associations and their implications for thyroid cancer management and prevention.﻿

## Conclusion

This study provides empirical evidence supporting the importance of the antioxidant system in the development and progression of mPTC and PTC. The increased concentration of TOS in mPTC and PTC compared to Healthy individuals confirms the pathogenic impact of oxidants. On the other hand, the lower level of GSH in mPTC patients compared to PTC, MNG, and Healthy individuals suggests a heightened presence of oxidative stress in these patients. In the case of mPTC, GSH could potentially be used as a diagnostic tumor marker. Further investigations are necessary to validate these findings and explore the potential therapeutic benefits of targeting the antioxidant system for the treatment of mPTC and PTC.

## Data Availability

The data that support this study are available from the corresponding author upon reasonable request.

## References

[1] Lorusso L, Cappagli V. Thyroid cancers: from surgery to current and future systemic therapies through their molecular identities. 2021;22(6). 10.3390/ijms22063117 PMC8003273 33803747

[2] Razavi SA, Salehipour P, Gholami H, Sheikholeslami S, Zarif-Yeganeh M, Yaghmaei P (2021). New evidence on tumor suppressor activity of PTEN and KLLN in papillary thyroid carcinoma. Pathol Res Pract.

[3] Hedinger C, Williams ED, Sobin LH (1989). The WHO histological classification of thyroid tumors: a commentary on the second edition. Cancer.

[4] Dideban S, Abdollahi A, Meysamie A, Sedghi S, Shahriari M (2016). Thyroid papillary Microcarcinoma: etiology, clinical manifestations,diagnosis, Follow-up, histopathology and prognosis. Iran J Pathol.

[5] Didehban S, Abdollahi A, Meysamie A (2023). Evaluation of etiology, clinical manifestations, diagnosis, Follow-up, histopathology and prognosis factors in papillary thyroid microcarcinoma: a systematic review and Meta-analysis. Iran J Pathol.

[6] Liu Q, Song M, Zhang H (2024). Choice of management strategy for papillary thyroid microcarcinoma: active surveillance or immediate surgery?. J Cancer.

[7] Amin MB, Greene FL, Edge SB, Compton CC, Gershenwald JE, Brookland RK (2017). The Eighth Edition AJCC Cancer staging Manual: continuing to build a bridge from a population-based to a more personalized approach to cancer staging. Cancer J Clin.

[8] Lucandri G, Fiori G, Falbo F, Pende V, Farina M, Mazzocchi P (2024). Papillary thyroid Microcarcinoma: differences between lesions in Incidental and Nonincidental settings-considerations on these clinical entities and personal experience. Current oncology (Toronto. Ont).

[9] Christofer Juhlin C, Mete O. The 2022 WHO classification of thyroid tumors: novel concepts in nomenclature and grading. 2023;30(2). 10.1530/ERC-22-0293 36445235

[10] Baloch ZW, Asa SL. Overview of the 2022 WHO Classification of Thyroid Neoplasms. 2022;33(1):27–63. 10.1007/s12022-022-09707-3 35288841

[11] Haugen BR, Sawka AM, Alexander EK, Bible KC, Caturegli P, Doherty GM (2017). American Thyroid Association Guidelines on the management of thyroid nodules and differentiated thyroid Cancer Task Force Review and recommendation on the proposed renaming of encapsulated follicular variant papillary thyroid Carcinoma without Invasion to Noninvasive follicular thyroid neoplasm with Papillary-Like Nuclear features. Thyroid: Official J Am Thyroid Association.

[12] Jelic MD, Mandic AD, Maricic SM, Srdjenovic BU (2021). Oxidative stress and its role in cancer. J Cancer Res Ther.

[13] Birben E, Sahiner UM, Sackesen C, Erzurum S, Kalayci O (2012). Oxidative stress and antioxidant defense. World Allergy Organ J.

[14] Pisoschi AM, Pop A (2015). The role of antioxidants in the chemistry of oxidative stress: a review. Eur J Med Chem.

[15] Wu G, Fang YZ, Yang S, Lupton JR, Turner ND (2004). Glutathione metabolism and its implications for health. J Nutr.

[16] Averill-Bates DA (2023). The antioxidant glutathione. Vitam Horm.

[17] Giustarini D, Colombo G, Garavaglia ML, Astori E, Portinaro NM, Reggiani F (2017). Assessment of glutathione/glutathione disulphide ratio and S-glutathionylated proteins in human blood, solid tissues, and cultured cells. Free Radic Biol Med.

[18] Traverso N, Ricciarelli R, Nitti M, Marengo B, Furfaro AL, Pronzato MA (2013). Role of glutathione in cancer progression and chemoresistance. Oxidative Med Cell Longev.

[19] Wu R, Feng J, Yang Y, Dai C, Lu A, Li J (2017). Significance of serum total Oxidant/Antioxidant status in patients with. Colorectal Cancer.

[20] Wang D, Feng JF, Zeng P, Yang YH, Luo J, Yang YW (2011). Total oxidant/antioxidant status in sera of patients with thyroid cancers. Endocrine-related Cancer.

[21] Buczyńska A, Sidorkiewicz I, Kościuszko M, Adamska A, Siewko K, Dzięcioł J (2023). Clinical significance of oxidative stress markers as angioinvasion and metastasis indicators in papillary thyroid cancer. Sci Rep.

[22] Buczyńska A, Sidorkiewicz I. The relationship between oxidative status and Radioiodine Treatment qualification among Papillary. Thyroid Cancer Patients. 2023;15(9). 10.3390/cancers15092436 PMC10177082 37173902

[23] Rovcanin BR, Gopcevic KR, Kekic D, Zivaljevic VR, Diklic A, Paunovic IR (2016). Papillary thyroid carcinoma: a malignant tumor with increased antioxidant defense capacity. Tohoku J Exp Med.

[24] Faam B, Ghadiri AA, Ghaffari MA, Totonchi M, Khorsandi L (2021). Comparing oxidative stress status among Iranian males and females with malignant and non-malignant thyroid nodules. Int J Endocrinol Metabolism.

[25] Acevedo-León D, Monzó-Beltrán L, Gómez-Abril S, Estañ-Capell N, Camarasa-Lillo N, Pérez-Ebri ML et al. The effectiveness of glutathione Redox Status as a possible tumor marker in Colorectal Cancer. 2021;22(12). 10.3390/ijms22126183 PMC8226858 34201191

[26] Rostami R, Nourooz-Zadeh S, Mohammadi A, Khalkhali HR, Ferns G, Nourooz-Zadeh J. Serum Selenium Status and Its Interrelationship with Serum Biomarkers of Thyroid Function and Antioxidant Defense in Hashimoto’s Thyroiditis. Antioxid (Basel Switzerland). 2020;9(11). 10.3390/antiox9111070 PMC7692168 33142736

[27] Razavi SA, Mahmanzar M, Nobakht M, Gh BF, Zamani Z, Nasiri S, Hedayati M. Plasma metabolites analysis of patients with papillary thyroid cancer: a preliminary untargeted 1H NMR-based metabolomics. J Pharm Biomed Anal. 2023:115946. 10.1016/j.jpba.2023.115946 38241910

[28] Marotta V, Russo G, Gambardella C, Grasso M, La Sala D, Chiofalo MG, D'Anna R, Puzziello A, Docimo G, Masone S, Barbato F, Colao A, Faggiano A, Grumetto L. Human exposure to bisphenol AF and diethylhexylphthalate increases susceptibility to develop differentiated thyroid cancer in patients with thyroid nodules. Chemosphere. 2019 Mar;218:885-894. doi: 10.1016/j.chemosphere.2018.11.084. Epub 2018 Nov 17. PMID: 30609493. 10.1016/j.chemosphere.2018.11.084 30609493

[29] Marotta V, Grumetto L, Neri I, Russo G, Tortora A, Izzo G, Panariello I, Rocco D, Pezzullo L, Vitale M. Exposure to Bisphenol A increases malignancy risk of thyroid nodules in overweight/obese patients. Environ Pollut. 2023 Jan 1;316(Pt 1):120478. doi: 10.1016/j.envpol.2022.120478. Epub 2022 Oct 25. PMID: 36306887. 10.1016/j.envpol.2022.120478 36306887

